# Vaccines for Cholera Control: Does Herd Immunity Play a Role

**DOI:** 10.1371/journal.pmed.0040331

**Published:** 2007-11-27

**Authors:** Lorenz von Seidlein

## Abstract

The author discusses a new study that mathematically simulated different vaccine coverage levels in the Matlab region of Bangladesh using a historic vaccine trial dataset.

## Disease Burden

Cholera is a diarrhoeal disease caused by *Vibrio cholerae* O1 and O139, transmitted through the faeco-oral route. The disease occurs in outbreaks but can establish itself permanently. The full impact of the disease is difficult to assess. The currently preferred measure of disease burden, disability-adjusted life years, fails to capture the enormous impact of a cholera outbreak, which spares no age group and paralyses the economy in severely affected areas.

The seventh cholera pandemic began in Indonesia in 1961 and spread quickly to other Asian countries, which became the epicentre of cholera outbreaks. With the economic emergence of Asia the number of cholera cases reported from that region has decreased. There are several possible reasons to explain this decline. First, massive investment has been made in providing a safe water supply and in sanitation. Second, reporting of cholera has become less reliable, because global trade—especially trade in seafood—and tourism are negatively affected by cholera outbreak reports.

In 1970 *Vibrio cholerae* O1 El Tor invaded sub-Saharan Africa, which had not experienced cholera for more than 100 years. In 2006, Africa reported 234,349 cases of cholera to the World Health Organization (WHO), accounting for 99% of the officially notified global cholera [1]. Between 1995 and 2005, 66% of cholera outbreak reports to ProMedmail (a global electronic reporting system for outbreaks of emerging infectious diseases and toxins, run by the International Society for Infectious Diseases, at http://www.promedmail.org/) came from sub-Saharan Africa [2]. There is growing evidence of the large and increasing burden of cholera in Africa.

**Linked Research Article**

This Perspective discusses the following new study published in *PLoS Medicine:*

Longini IM, Nizam A, Ali M, Yunus M, Shenvi N (2007) Controlling endemic cholera with oral vaccines. PLoS Med 4(11): e336. doi:10.1371/journal.pmed.0040336

Using data from Bangladesh, Ira Longini and colleagues develop a mathematical model predicting that oral vaccination of 50%–70% of the population could control cholera transmission in an endemic region.

Most recently the US-led invasion of Iraq has been accompanied by a re-emergence of cholera in that country. As of September 2007, nearly 7,000 cholera cases from the Sulaymaniyah and Kirkuk Governates have been reported to WHO [3].

## Cholera Control and Vaccines

Cholera was eliminated from the industrialized world through safer water supplies, better sanitation, and improved food hygiene. These have been the accepted control mechanisms for the disease, but as the emergence of cholera in Iraq illustrates, the provision of safe water and sanitation breaks down during wars and complex humanitarian emergencies. In addition to these crisis situations, cholera also thrives in the ever-increasing slums of some megacities such as Kolkata (formerly Calcutta), India, which are not quickly accessible to improvements in infrastructure.

In 2002, WHO mentioned for the first time the potential use of oral cholera vaccines in endemic and epidemic situations [4]. Up to that point cholera vaccines were recommended for individual travellers to endemic countries but not for public health use in endemic countries. Far from embracing vaccinations for cholera control, WHO experts recommended gaining more experience through demonstration projects. Since then, mass oral cholera vaccinations have been conducted in Beira, Mozambique, in Darfour, Sudan, and in Aceh, Indonesia. These projects demonstrated the feasibility and effectiveness of vaccination under actual public health conditions [5]. A WHO meeting at the end of 2005 suggested that “… the use of oral cholera vaccines in certain endemic situations should be recommended…” [6].

The slow acceptance of vaccines for cholera control is probably related to the poor performance of earlier generations of cholera vaccines made from phenol-killed whole-cell preparations of *V. cholerae* O1 organisms and administered by injection as two doses, two weeks apart. The vaccine offered about 50% protection for a short duration, was associated with painful local inflammatory reactions, and is no longer recommended for use [7].

Currently two cholera vaccines are internationally licensed: (1) Dukoral, consisting of inactivated whole cells of *V. cholerae* O1 combined with the B-subunit (BS-WC) of the cholera toxin, and (2) the live attenuated vaccine CVD 103HgR (Orochol or Mutacol). Both vaccines have an excellent safety profile and afford high rates of protection over several years. The companies producing each vaccine have been acquired in the last two years by the publicly listed Dutch company Crucell. Only Dukoral is currently produced, and for this reason was used in the above-mentioned vaccination campaigns in Mozambique, Sudan, and Indonesia. Dukoral costs travellers more than US$10 per dose, and two doses two weeks apart are recommended for immunisation. Most tourists can afford this vaccine, and well-supported foundations can purchase this vaccine for interventions in larger populations. Yet Dukoral is likely to remain too expensive for governments of cholera-endemic regions to vaccinate at-risk populations. The technology on which Dukoral is based has previously been transferred to manufacturers in Vietnam, and has been more recently transferred to an Indian vaccine producer with certification to produce internationally licensed vaccines. There is therefore a justified hope that this vaccine candidate will become available internationally at an affordable price.

The ideal cholera vaccine is safe and affords extended if not lifelong protection after a single dose. It can be stored for extended periods at room temperature and is in the same price range as vaccines included in the WHO Expanded Program on Immunization. Promising candidates approaching this ideal are under development. Peru-15, for example, is a live, attenuated vaccine candidate that has been found to be safe and immunogenic in infants and children in Bangladesh [8,9]. Because cold storage presents a challenge for the use of this vaccine in tropical cholera endemic regions, a thermostable vaccine is under development. A promising live, attenuated cholera vaccine candidate is being developed by the Cuban Finley Institute (Camaguey, Cuba); this candidate is currently under evaluation in sub-Saharan Africa and could become available at an affordable price [10].

## Herd Protection Conferred By Oral Cholera Vaccines

With the current availability of one vaccine, the development of better candidates, and the endorsement of WHO, wider use of vaccines for cholera control looks promising. From a policy maker's perspective, it would be useful to know the level of vaccine coverage to aim for to control cholera in a community, and second, whether this approach is cost-effective.

Recent evidence for herd protection conferred by oral cholera vaccinations suggests that immunising a fraction of a community reduces the transmission of cholera sufficiently for the unvaccinated members of the community to benefit from reduced risk of disease [11]. However, while it has become clear that oral cholera vaccine programs will be more cost-effective than previous trial data had suggested, the level of coverage required to control cholera remains unknown.

In a study published in this issue of *PLoS Medicine*, Ira Longini and colleagues [12] have mathematically simulated varying vaccine coverage levels in the Matlab region of Bangladesh using a historic vaccine trial dataset [13,14]. Their simulations suggest that in a population in which 50% received an oral cholera vaccine, 93% of the overall population would be protected; this level of protection, the authors think, would result in control of cholera transmission. To make their model more generalisable, they modified the assumptions in a sensitivity analysis. For populations that have less natural immunity than Matlab, 70% coverage would likely be necessary to provide sufficient protection to control cholera.

If the authors are correct, an achievable goal of 50% vaccine coverage could protect high-risk populations suffering recurrent cholera outbreaks. This suggestion is highly encouraging, because 50% vaccine coverage has been achieved in earlier mass vaccination campaigns. Current problems in eradication of poliovirus illustrate how difficult it is to reach the last 10% of the population to drive coverage above 90%. Reaching only half of the population is comparatively easy.

## What Remains to be Done?

The findings of Longini and colleagues are encouraging, but policies tend to rely on actual field-derived data, not on models. The authors had access to one dataset from a trial conducted more than 20 years ago in the Bay of Bengal, a place considered at the time by cholera experts as the “home of cholera,” probably not representative for the global cholera situation in 2007. More data from a variety of settings is now needed.

Based on the models provided by Longini and colleagues, we would expect that mass vaccination campaigns with reasonable coverage in isolated areas with stable populations should eliminate cholera for several years. In contrast, vaccination campaigns in urban slums with highly mobile populations could have a lower impact. Nothing is known about the impact of a combination of improved water supplies and sanitation with vaccination campaigns, which could be additive or synergistic. The key to a better understanding is close documentation of interventions and outcomes, which will allow us to confirm or reject Longini and colleagues' models.

## Abbreviations

WHO: World Health Organization
